# Palliative Interventional and Surgical Therapy for Unresectable Pancreatic Cancer

**DOI:** 10.3390/cancers3010652

**Published:** 2011-02-14

**Authors:** Volker Assfalg, Norbert Hüser, Christoph Michalski, Sonja Gillen, Jorg Kleeff, Helmut Friess

**Affiliations:** Department of Surgery, Klinikum rechts der Isar, Technische Universität München, Ismaningerstr. 22, D-81675 Munich, Germany; E-Mails: assfalg@chir.med.tu-muenchen.de (V.A.); hueser@chir.med.tu-muenchen.de (N.H.); michalski@chir.med.tu-muenchen.de (C.M.); gillen@chir.med.tu-muenchen.de (S.G.); kleeff@chir.med.tu-muenchen.de (J.K.)

**Keywords:** pancreatic cancer, palliative pancreatic surgery, neoadjuvant therapy, duodenal obstruction, jaundice, prophylactic gastroenterostomy

## Abstract

Palliative treatment concepts are considered in patients with non-curatively resectable and/or metastasized pancreatic cancer. However, patients without metastases, but presented with marginally resectable or locally non-resectable tumors should not be treated by a palliative therapeutic approach. These patients should be enrolled in neoadjuvant radiochemotherapy trials because a potentially curative resection can be achieved in approximately one-third of them after finishing treatment and restaging. Within the scope of best possible palliative care, resection of the primary cancer together with excision of metastases represents a therapeutic option to be contemplated in selected cases. Comprehensive palliative therapy is based on treatment of bile duct or duodenal obstruction for certain locally unresectable or metastasized advanced pancreatic cancer. However, endoscopic or percutaneous stenting procedures and surgical bypass provide safe and highly effective therapeutic alternatives. In case of operative drainage of the biliary tract (biliodigestive anastomosis), the prophylactic creation of a gastro-intestinal bypass (double bypass) is recommended. The decision to perform a surgical *versus* an endoscopic procedure for palliation depends to a great extent on the tumor stage and the estimated prognosis, and should be determined by an interdisciplinary team for each patient individually.

## 1. Introduction

Despite intensive research and therapeutic achievements in recent decades, the prognosis of pancreatic cancer has not improved significantly. The mean life expectancy for patients at all stages still remains six to eight months due to local progress of disease, lymphatic metastases, perineural invasion, or disseminated metastases at the time of diagnosis [1]. Besides conventional surgical methods, diverse alternatives with palliative intent could be established in recent years, such as drainage of the bile duct or removal of gastric or duodenal obstruction.

Nevertheless, the unequivocal resectability of the tumor can still be determined by operative exploration only. Radical operative procedures such as extended lymphatic dissection or segmental resection of the superior mesenteric artery have failed to demonstrate significant advantages in survival compared to standard methods [2,3]. In selected patients, a partial duodeno-pancreatectomy with R2 resection is under discussion as a therapeutic option, and the impact of resection of metastases continues to be a controversial topic. The high incidence of local recurrence due to microscopically incomplete resection (R1) and the intention to reduce the size of an initially unresectable tumor serve as strong arguments for multimodal neoadjuvant trials.

In cases of unresectability due to extensive tumor spread, percutaneous transhepatic cholangiography and biliary drainage (PTCD) is performed, when the transpapillary approach for an endoscopically introduced biliary drainage is not practicable to palliate malignant obstructive jaundice. Alternatively, highly effective operative procedures for biliary drainage can be applied.

In approximately 5–20% of patients with pancreatic cancer, infiltrative tumor expansion causes gastrointestinal obstruction. Although gastroenterostomy and hepaticojejunostomy are technically well established and safe operative procedures, only few studies have analyzed the impact of a prophylactic double bypass in case of intraoperative diagnosis of advanced pancreatic tumor disease. Whereas stenting of the biliary system requires periodic changes of the endoprosthesis and causes relevant hospitalization, a single surgical (palliative) procedure may allow longer-term quality of life [4]. Nevertheless, it is still a considerable clinical challenge to identify those patients who will receive maximum benefit from a bypass operation in view of their remaining time of life.

This article gives an overview of current therapeutic options for unresectable pancreatic cancer identified either pre- or intraoperatively. It outlines the potential benefit of resection for metastases, presents the option of neoadjuvant therapy within the scope of a multimodal therapeutic trial, displays the indications for endoscopically performed stenting and operative hepaticojejunostomy/gastroenterostomy, and reflects on the possible indications for a prophylactic double bypass and the relative importance of palliative surgery in the therapy of unresectable pancreatic cancer.

## 2. Surgery for Metastasized Pancreatic Cancer

Generally, evidence of metastasized pancreatic cancer is a contraindication for surgery because increasing morbidity and mortality associated with resection procedures outweigh potential benefits. However, a small group of patients might theoretically benefit from an extension of the defined resectability criteria [5-9]. Recently, a systematic analysis of the literature on M1 resections in pancreatic cancer patients was performed by our study group to clarify this controversial topic [10]. In this study, we found that in certain patients with M1 resections, surgery-associated morbidity is approximately 25% and mortality is very low (0–4%). Furthermore, median survival is between five and 11 months, demonstrating that patients who have undergone resection for metastases at least do not fare worse than patients receiving standard palliative treatment. Therefore, we concluded that metastasectomy in pancreatic cancer does not impose a greater operative risk than standard pancreatic resections. A rough estimation of the number of pancreatic cancer resections performed worldwide in the past 30 years (probably more than 150,000 cases) and a comparison to the number of published metastasectomy cases (103) showed that “the general concern should not be that in a few cases boundaries are crossed, but that in a much larger number, opportunities for prolonged survival and better quality of life are lost”. Because pancreatic cancer surgery is not curative in the majority of cases but still the best palliative treatment, resections for metastasized disease should be discussed as an option in the palliative therapeutic situation.

## 3. Neoadjuvant Therapy

Neoadjuvant therapy in pancreatic cancer has been investigated in numerous phase I and phase II trials over more than two decades [11]. Although its benefit in treating various other tumor entities has been shown in the past, the clinical advantage of neoadjuvant therapy in pancreatic cancer has not been proven so far [12]. Currently, there is a lack of controlled randomized studies, data on low response rates combined with relevant toxicity of pretreatment, and controversial reports on both resectability and survival after preoperative radiation and/or chemotherapy. In addition, assessment of the resectability criteria and evaluation of the response to pretreatment are highly dependent on the expertise of the involved surgeons, gastroenterologists, and radiologists [13].

In patients with marginally resectable tumors, neoadjuvant therapy may induce a *down-staging* and therefore possibly enable resectability or sufficient local tumor control [14]. The medical term *‘marginal resectability’* has been established because of inconsistent definitions of *resectability* and *unresectability*, which makes an efficient analysis of these cases quite difficult [13]. In a recent meta-analysis on neoadjuvant therapeutic trials in pancreatic cancer including approximately 4,400 patients with preoperative radiation and/or chemotherapy, the authors also differentiate between the group of patients with initially *resectable* pancreatic cancer on the one hand and a marginally resectable or unresectable tumor on the other hand [15]. For the group of patients with resectable tumors, an analysis of 111 studies revealed comparable results for resection in terms of median survival after neoadjuvant therapy (23.3 months) or immediate resection and subsequent adjuvant or additive therapy (median survival 20.1–23 months) [16-18]. Remarkably, in the group of patients with only marginally resectable or unresectable tumors, the neoadjuvant treatment increased the median survival rate to 20.5 months in a third of the cases, which is comparable to the group of primarily resectable tumors [15]. However, controlled randomized trials are particularly necessary in the group with initially unresectable tumors to establish optimal therapy protocols and to confirm the results of the above-mentioned meta-analysis. At present, only one controlled randomized study exists on neoadjuvant radio-chemotherapy *versus* immediate resection, and unfortunately it had to be stopped ahead of schedule because not enough patients could be recruited.

## 4. Endoscopically Performed Stenting and Operative Hepaticojejunostomy/Gastroenterostomy

Despite the development of modern interventional techniques for biliary drainage or deobstruction of an occluded gastroduodenal tract, therapy remains a matter of controversy, whether the ‘gold standard’ should be endoscopic, operative, or combined.

Only a few, mainly retrospective studies, compare bypass surgery and endoscopic stenting. The only prospectively performed and randomized study (18 patients) concerning gastric or duodenal obstruction due to progressive tumor infiltration and growth could not show any difference in the long-term outcome [19]. Retrospective analyses—regrettably including only small numbers of patients—revealed increased morbidity and procedure-related mortality, longer hospital stay [20], and a prolonged period until oral feeding [21] for operative gastrojejunostomy. A systematic review including more than 600 patients who underwent treatment with a self-expanding metal stent showed 0% procedure-related mortality and only 18% stent occlusion in the follow-up [22].

The primary target of palliation in patients with pancreatic or ampullary cancer with jaundice because of biliary obstruction is drainage using an endoscopically or percutaneously implanted stent or operative bypass. Deziel *et al.* prefer the interventional therapy only in patients with far advanced cancer and severely reduced physical well-being [23].

A meta-analysis comparing surgical biliary drainage (bypass operation) and endoscopically implanted biliary stents for malignant biliary obstruction, based on three randomized studies published between 1988 and 1994, revealed a frequency of 3% (0–16%) for surgical re-intervention and 36% (28–43%) for stent replacement due to dislocation or occlusion of the stent [24]. A British study (Sheperd *et al.* [25]) and a Danish study (Andersen *et al.* [26]) including high numbers of patients could not uncover any differences in complication rates. Only Smith *et al.* [27] reported a decreased percentage of complications (11% *vs.* 29%) and lower procedure-related mortality (3% v*ersus* 14%) in the endoscopically treated group. The study published by Andersen *et al.* showed significantly lower failure of therapy in interventionally treated patients.

A randomized, prospective study performed by van Dijkum *et al.* was based on laparoscopy to analyze 27 patients with pancreatic cancer who were categorized as ‘unresectable’ [28]. No procedure-related mortality was noted and morbidity was low. The average length of survival of patients who were randomized into the palliative endoscopic arm of the study (n = 14) was 116 days, whereas the mean survival of patients with surgical palliation (n = 13) was 192 days. In another study, Brandabur *et al.* reported comparable results [29] but a higher rate of hospitalization because of occluded or displaced stents. Despite higher morbidity, longer hospital stay, and increased mortality in patients who received bypass surgery, several other surveys demonstrated a better long-term outcome in this group [30-32].

Several reviews investigated short- and long-term outcome after palliative surgery for biliary drainage, such as cholecysto- or hepaticojejunostomy. Sarr and Cameron examined publications on this topic including a total number of more than 8,000 patients with pancreatic carcinoma between 1965 and 1980. They verified an operative morbidity of 16% for cholecystojejunostomy and 20% for hepaticojejunostomy. In the cholecystojejunostomy group, a recurrence of jaundice with consecutive cholangitis occurred in up to 10% of cases, whereas this was not seen in the hepaticojejunostomy group, but the difference was not significant [33]. At least since a comprehensive meta-analysis by Watanapa *et al.*, hepaticojejunostomy has become the standard surgical procedure (success quotient 97% *versus* 89% for cholecystoenterostomy) [34].

The PTCD is used in those cases in which the tumor size renders an endoscopic approach to the papilla or an endoscopic stent implantation into the biliary tract impossible. To date, only one prospective study compared PTCD and an operative bypass procedure [35], revealing an increased rate of recurrence of jaundice in patients treated by non-operative intervention. Recently, Bornman *et al.* presented data showing no major differences in survival, hospital stay, or complication rate between patients who received either operative hepaticojejunostomy or percutaneously implanted endoprothesis [36].

Currently there are no studies comparing interventional stenting and laparoscopically performed bypass.

To facilitate the decision for operative or interventional therapy, independent prognostic factors such as the ASA score, evidence of liver metastases, or certain levels of tumor markers (e.g., CA19-9, CEA) may help in creating an interdisciplinary therapy plan. In a multivariate data analysis these factors enabled differentiation between short-term and long-term survivors [37].

## 5. Prophylactic Double Bypass and the Importance of Palliative Surgery in the Therapy of Unresectable Pancreatic Cancer

In cases when a pancreatic tumor is determined to be unresectable during the operation, the surgeon can easily perform a double bypass, consisting of a biliodigestive anastomosis and gastrojejunostomy. The intraoperative decision to perform prophylactic gastrojejunostomy remains challenging, not the least because of the difficulty of identifying those patients who are most likely to develop pyloroduodenal obstruction because of advanced tumor disease. After surgery for biliodigestive anastomosis only, 20%–30% of the patients show pyloroduodenal obstruction, nausea, and vomiting in the follow-up [34,38].

Three major reviews evaluated the status of prophylactic gastrojejunostomy in unresectable pancreatic cancer [30,34,38]: An analysis of more than 3,300 cases after palliative biliodigestive anastomosis (data from literature published between 1965 and 1983) revealed a re-intervention rate of 16% due to duodenal compression. Furthermore, the review could not determine an elevated operative mortality in case of simultaneously performed gastroenterostomy (13% *versus* 14%) [30]. A review by Watanapa *et al.*, including more than 1,600 patients treated between 1973 and 1990, confirmed the necessity of operative gastrojejunostomy in 17% of the cases and validated that mortality after double bypass surgery is not elevated [34]. The authors therefore preferred an additional gastroenterostomy to preempt duodenal obstruction in the case of unresectable pancreatic cancer when assessed during explorative laparotomy. Moreover, they found increased morbidity when a second operation was necessary. Furthermore, a review on 950 patients identified the need for gastroenterostomy in up to 21% [38]. Huguier *et al.* and Neuberger *et al.* recommended simultaneous biliary and enteral bypasses on the basis of comparable mortality (single *versus* double bypass), but found an increased need for gastric bypass at a later date (when most patients' physical condition has deteriorated) in patients who initially only received biliary drainage [39,40]. This approach is supported by the results of several other studies [41-44]. On the contrary, other authors emphasize in particular the selective aspect of performing a double bypass in patients with advanced pancreatic cancer. In the face of this aggressive and rapidly progressive tumor entity, routine implementation of an additional gastroenterostomy seems to be redundant [33,35,45,46].

Currently, only two prospective studies analyzing a possibly existing advantage of prophylactic double bypass are available. The work of Lillemoe *et al.* included 87 patients with an unresectable lesion of the periampullary region but without signs of duodenal obstruction. Beyond hepaticojejunostomy, patients received either additional retrocolic gastrojejunostomy or no prophylactic enteral bypass [47]. In both groups the total postoperative morbidity rate (32% *versus* 33% in the group without gastrojejunostomy) and the length of hospital stay (8.5 ± 0.5 days *versus* 8.0 ± 0.5 days) were comparable. The investigations mainly revealed an advantage in long-term surveillance, as 19% of the patients who underwent only surgery for biliary drainage developed intestinal obstruction. In addition, postoperative delayed gastric emptying (DGE) appeared with the same frequency (2%) in both groups. Furthermore, the authors of a randomized prospective study from the Netherlands strongly recommended a routinely performed prophylactic double bypass in case of diagnosis of unresectable pancreatic cancer during explorative laparotomy. The same study identifies postoperative DGE as the main side effect of prophylactic gastroenterostomy (17%, *versus* 3% after biliary bypass only). Nevertheless, this procedure leads to a significantly decreased rate of gastric outlet obstruction and less need for relaparotomy (5.5% *versus* 41.4%) [48].

A prospective analysis by Shyr *et al.* concluded that prophylactic gastrojejunostomy (in addition to biliary bypass) appears to be quite advantageous, because more than 30% of the patients with single bypass develop obstructive gastric emptying disorders, whereas in the patients undergoing prophylactic gastrojejunostomy, postoperative morbidity and mortality are not increased [49]. A meta-analysis on this topic including a total of 218 patients revealed a significantly lower incidence of obstructive gastric emptying disorders in patients with double bypass, although postoperative DGE was comparable. Furthermore, both groups showed no difference in morbidity and mortality [50].

## 6. Summary

As surgery for pancreatic cancer is non-curative in many cases, resection of metastases provides an additional option for palliative therapy in selected cases.

According to the latest data, patients with initially unresectable, non-metastasized pancreatic cancer should be recruited into neoadjuvant radiochemotherapy trials. After finishing neoadjuvant therapy, resection can be performed in as many as one-third of the patients.

The majority of patients undergo implantation of a large-bore plastic or expanding biliary stent by endoscopic retrograde cholangioscopy (ERC). Biliary drainage via PTCD is rarely necessary. Alternatively, a biliodigestive anastomosis and gastroenterostomy should be considered as a first-line therapeutic option.

Even though prophylactic gastroenterostomy is a safe surgical procedure, the operative findings and the next therapeutic steps should be considered carefully by the surgeon, because gastroenterostomy remains a therapy which is restricted to a selected group of patients dependent on life expectancy.
